# 

**Published:** 1849-04

**Authors:** 


### TO CORRESPONDENTS AND PUBLISHERS.

The communications of Drs. Buchanan, Dawson, Barnett, and Haskins have been received, for which the authors will please accept our acknowledgments. They will appear as early as room can be found for them. We are indebted to publishers for a number of new works which will be noticed at the earliest practicable day.

### RECEIPTS OF MEDICAL JOURNAL FOR MARCH 1849.

| | |
|---|---|
| Dr. Wharton, Nashville, Tenn. | $ 5 00 |
| R. Atkisson, Simpsonville, Ky. | 5 00 |
| —— Porter, Jacksonville, Ala. | 5 00 |
| A. McBean, Metropolis City, Ill. | 5 00 |
| —— McLurkin, Halselville, S. C, | 5 00 |
| F. J. Yager, County. | 2 50 |
| D. Drake, Cincinnati, O. | 5 00 |
| W. Buckner, Georgetewn, O. | 5 00 |
| C. C. Smith, County. | 5 00 |
| R. W. Washington, County. | 5 00 |
| C. R. McGill, Elizabethtown, Ky. | 5 00 |
| J. Z Wheat, Columbia, Ky. | 5 00 |
| —— Alsup, Statesville, Tenn. | 5 00 |
| T. C. Osborne, Erie, Ala. | 20 00 |
| W. R. Winton, Somerville, O. | 2 00 |
| R. Steele, Paris. Ill. | 5 00 |
| R. A. Westbrook, Oakland, Tenn. | 10 00 |
| J. D. McGill, Burkettsville, Miss. | 2 00 |

The WESTERN JOURNAL OF MEDICINE and SURGERY is published monthly by the undersigned, at the corner of Main and Fifth streets, Louisville, at $5 per annum, payable in advance. Each number contains 96 pages, making two volumes in the year of more than 550 pages each.

Contributions to its pages by the Physicians of the Valley of the Mississippi, addressed to the Editors, are respectfully solicited.

[illegible] business to be addressed, postage paid to the publishers.

Jauuary, 1844 PRENTICE & WEISSINGER.

